# Short Tandem Repeat Variation in the *CNR1* Gene Associated With Analgesic Requirements of Opioids in Postoperative Pain Management

**DOI:** 10.3389/fgene.2022.815089

**Published:** 2022-03-03

**Authors:** Shinya Kasai, Daisuke Nishizawa, Junko Hasegawa, Ken-ichi Fukuda, Tatsuya Ichinohe, Makoto Nagashima, Masakazu Hayashida, Kazutaka Ikeda

**Affiliations:** ^1^ Addictive Substance Project, Department of Psychiatry and Behavioral Sciences, Tokyo Metropolitan Institute of Medical Science, Tokyo, Japan; ^2^ Department of Oral Health Science, Tokyo Dental College, Tokyo, Japan; ^3^ Department of Dental Anesthesiology, Tokyo Dental College, Tokyo, Japan; ^4^ Department of Surgery, Toho University Sakura Medical Center, Sakura, Japan; ^5^ Department of Anesthesiology and Pain Medicine, Juntendo University School of Medicine, Tokyo, Japan

**Keywords:** analgesic requirement, cannabinoid receptor 1, ionotropic glutamate receptor NMDA type subunit 2A, preproenkephalin, prodynorphin, short tandem repeat

## Abstract

Short tandem repeats (STRs) and variable number of tandem repeats (VNTRs) that have been identified at approximately 0.7 and 0.5 million loci in the human genome, respectively, are highly multi-allelic variations rather than single-nucleotide polymorphisms. The number of repeats of more than a few thousand STRs was associated with the expression of nearby genes, indicating that STRs are influential genetic variations in human traits. Analgesics act on the central nervous system via their intrinsic receptors to produce analgesic effects. In the present study, we focused on STRs and VNTRs in the *CNR1*, *GRIN2A*, *PENK*, and *PDYN* genes and analyzed two peripheral pain sensation-related traits and seven analgesia-related traits in postoperative pain management. A total of 192 volunteers who underwent the peripheral pain sensation tests and 139 and 252 patients who underwent open abdominal and orthognathic cosmetic surgeries, respectively, were included in the study. None of the four STRs or VNTRs were associated with peripheral pain sensation. Short tandem repeats in the *CNR1*, *GRIN2A*, and *PENK* genes were associated with the frequency of fentanyl use, fentanyl dose, and visual analog scale pain scores 3 h after orthognathic cosmetic surgery (Spearman’s rank correlation coefficient *ρ* = 0.199, *p* = 0.002, *ρ* = 0.174, *p* = 0.006, and *ρ* = 0.135, *p* = 0.033, respectively), analgesic dose, including epidural analgesics after open abdominal surgery (*ρ* = −0.200, *p* = 0.018), and visual analog scale pain scores 24 h after orthognathic cosmetic surgery (*ρ* = 0.143, *p* = 0.023), respectively. The associations between STRs in the *CNR1* gene and the frequency of fentanyl use and fentanyl dose after orthognathic cosmetic surgery were confirmed by Holm’s multiple-testing correction. These findings indicate that STRs in the *CNR1* gene influence analgesia in the orofacial region.

## Introduction

Pain sensation is an inherent mechanism to avoid tissue damage from injuries and burns. It is an essential system for the living body, but excessive pain is a harmful sensation that evokes unpleasant and aversive reactions. Therefore, there is a clinical need to adequately control severe pain. Opioids, including morphine and fentanyl, have been used as analgesics to alleviate severe pain in many medical situations, such as cancer pain treatment and postoperative pain management. Opioid analgesic consumption varies widely among countries. Palliative care has been reported to be practiced adequately or moderately in the United States, Canada, Europe, and Australia (Duthey and Scholten, 2014). However, in recent years, opioid use disorder, including opioid misuse and addiction, has caused a serious public health crisis in these countries (Volkow and Collins, 2017). The morbidity and mortality of opioid use disorder increased during the coronavirus disease 2019 pandemic in the United States and Canada. Thus, proper opioid use is required now more than ever (Patel et al., 2021; Wang et al., 2021).

A great deal of variation in opioid requirements has been observed for adequate pain relief among individuals (Aubrun et al., 2003; Fukuda et al., 2010). Individual differences in opioid analgesia are attributable to multiple and interacting genetic, psychological, and environmental factors. Twin studies that compared morbidity between monozygotic and dizygotic twins reported that 12–60, 30, 59, and 36% were heritable for opioid-induced analgesic effects, respiratory depression, nausea, and drug dislike, respectively (Angst et al., 2012a; Angst et al., 2012b). Recently, genetic studies of personalized pain treatment have been performed, and some genetic variations have been shown to be associated with individual differences in analgesic effects of opioids (reviewed in Kumar et al., 2019). The A118G single nucleotide polymorphism (SNP; rs1799971) in the μ-opioid receptor (*OPRM1*) gene is the most studied genetic variation that is associated with individual differences in opioid analgesia (Kasai and Ikeda, 2011). Single-nucleotide polymorphisms in the ATP-binding cassette subfamily B member 1 (*ABCB1*), adrenoceptor β1 (*ADRB1*), catechol-*O*-methyltransferase (*COMT*), cytochrome P450 family 2 subfamily D member 6 (*CYP2D6*), cytochrome P450 family 3 subfamily A member 4 (*CYP3A4*), solute carrier family 22 member 1 (*SLC22A1*), and UDP glucuronosyltransferase family 2 member B7 (*UGT2B7*) genes were also shown to be associated with analgesic effects of opioids (Reyes-Gibby et al., 2007; Stamer et al., 2007; Rakvåg et al., 2008; Tzvetkov et al., 2011; Sistonen et al., 2012; Candiotti et al., 2013; Bastami et al., 2014; Dong et al., 2015; Wei et al., 2015; Dzambazovska-Trajkovska et al., 2016; Lv et al., 2018).

Besides SNPs, various types of genetic variations, including insertions and deletions (i.e., indels), short tandem repeats (STRs), variable number of tandem repeats (VNTRs), copy number variations (CNVs), and retrotransposons, exist in the human genome. Short tandem repeats and VNTRs are typically classified as repetitive sequence motifs of less than six base pair (bp) nucleotides and more than six bp, respectively. Short tandem repeats and VNTRs were identified in the human genome at approximately 0.7 and 0.5 million loci, respectively, which are less than the 85 million loci for SNPs (Willems et al., 2014; Genomes Project Consortium et al., 2015; Bakhtiari et al., 2021). However, repeat variations of STRs vary widely because of the high mutation rate by DNA slippage (Fan and Chu, 2007). The rate of mutation of STRs in the human germline was estimated to be approximately 3–7 × 10^–3^ per locus per gamete per generation (Brinkmann et al., 1998; Ellegren, 2000; Kayser et al., 2000). The genome-wide mutation rate in the human germline is relatively low compared with STRs (approximately 10^–8^ to 10^–9^ per locus per generation; Scally, 2016; Xue et al., 2009). VNTRs are repeat sequences of approximately 10–100 nucleotides with 5–50 repeats and the large nucleotide differences would affect chromosomal structures (Brookes, 2013). Therefore, STRs and VNTRs highly contribute to the heritability of individual differences in human traits.

Short tandem repeats and VNTRs in the cannabinoid receptor 1 (*CNR1*), glutamate ionotropic receptor NMDA type subunit 2A (*GRIN2A*), prodynorphin (*PDYN*), and proenkephalin (*PENK*) genes were reported to be associated with the vulnerability to schizophrenia and substance dependence (Comings et al., 1997; Comings et al., 1999; Chen et al., 2002; Itokawa et al., 2003). Opioid analgesics act through opioid receptors, which mediate almost all opioid effects and comprise the endogenous opioid system with their endogenous peptide ligands, including enkephalins and dynorphins. The *PDYN* and *PENK* genes encode prodynorphin and preproenkephalin, which are precursors of dynorphin and enkephalin peptides, respectively. The *CNR1* gene encodes cannabinoid receptor 1, which is mainly expressed in the central and peripheral nervous systems, including the hippocampus, frontal cortex, amygdala, hypothalamus, cerebellum, and midbrain (Glass et al., 1997). The endocannabinoid system via CNR1 was reported to interact closely with the endogenous opioid system. The allosteric modulation of CNR1 signaling altered opioid function, including antinociception, in rodents (Datta et al., 2020; Slivicki et al., 2020). The *GRIN2A* gene encodes the ionotropic glutamate receptor NMDA2A (ε1), which is a subunit of NMDA receptor channels. *GRIN2A* gene expression is abundant in the central nervous system and increases during development in humans and mice (Bagasrawala et al., 2017; Rodenas-Ruano et al., 2012; Sanz-Clemente et al., 2010, Sanz-Clemente et al., 2013). Opioid effects, including tolerance and reward, were reduced in *Grin2a* knockout mice (Miyamoto et al., 2004).

In the present study, STRs and VNTR in the *CNR1*, *GRIN2A*, *PDYN*, and *PENK* genes were examined to assess genetic vulnerability to peripheral pain sensation and analgesic requirements for opioids, based on the latency to cold or mechanical pain perception, frequency of fentanyl and analgesic use, dose of fentanyl and analgesic use, and visual analog scale (VAS) score after surgery in postoperative pain management.

## Materials and Methods

### Participants

The present study was conducted with clinical data from 192 participants to assess peripheral pain sensation, 139 patients who underwent major abdominal surgery, and 252 patients who underwent orthognathic cosmetic surgery. Volunteers who lived in the Kanto area of Japan were enrolled in the study to assess peripheral pain sensation. The patients who underwent major open abdominal surgery with combined general and epidural anesthesia were enrolled at the Research Hospital, Institute of Medical Science, The University of Tokyo, or Toho University Sakura Hospital. Patients who were scheduled to undergo orthognathic cosmetic surgery (bilateral mandibular sagittal split ramus osteotomy) for mandibular prognathism were enrolled at Tokyo Dental College Suidoubashi Hospital. All of the subjects provided written informed consent before participating in the study.

The study protocol was approved by each Institutional Review Board at the Institute of Medical Science, The University of Tokyo (Tokyo, Japan), Toho University Sakura Hospital (Chiba, Japan), Tokyo Dental College (Chiba, Japan), and the Tokyo Metropolitan Institute of Medical Science (Tokyo, Japan).

### Clinical Data on Peripheral Pain Sensation and Analgesic Requirements of Opioids

In the study of peripheral pain sensation, latencies to cold and mechanical pain perception were measured in the cold pressor- and mechanical stimulation-induced pain tests, respectively. The cold pressor-induced pain test was slightly modified from original methods (Bisgaard et al., 2001; Martikainen et al., 2004). Briefly, each index and middle finger was placed in ice-cold water up to the second joint, and the time to feel a pain sensation was measured with a 15 s cut-off time to prevent tissue damage. The average of the two fingers was considered the latency to cold pain perception. A DPS-20 digital force gauge (Imada, Northbrook, IL, United States) was used in the mechanical stimulation-induced pain test. The nail of each index finger to pinky finger was pressed with the digital force gauge, and the force to feel pain sensation was recorded. The average of the four fingers was considered the latency to mechanical pain perception (Nishizawa et al., 2014).

Postoperative pain management after open abdominal surgery was previously described (Hayashida et al., 2008). Briefly, postoperative pain management was primarily performed with continuous epidural anesthesia with fentanyl or morphine. For patients who complained of significant postoperative pain despite the administration of continuous epidural analgesics, appropriate doses of opioids, including morphine, buprenorphine, pentazocine, and pethidine, and/or nonsteroidal antiinflammatory drugs (NSAIDs), including diclofenac and flurbiprofen, were systemically administered as rescue analgesics and antipyretics. The doses of opioids and NSAIDs that were administered as rescue analgesics during the postoperative period were converted to equivalent doses of systemic pentazocine. The frequency of rescue analgesic use during the 24 h postoperative period, total dose of rescue analgesics during the 24 h postoperative period (converted to equivalent doses of systemic pentazocine), and total dose of rescue analgesics, excluding antipyretics, during the 24 h postoperative period (converted to equivalent doses of systemic pentazocine) were calculated and analyzed.

The clinical data on postoperative pain management after orthognathic cosmetic surgery were previously reported (Fukuda et al., 2009). Briefly, intravenous patient-controlled analgesia (PCA) with 20 μg/ml fentanyl began immediately after tracheal extubation using a CADD-Legacy PCA pump (Smiths Medical Japan, Tokyo, Japan). A bolus dose of fentanyl (20 μg) was administered upon patient request. Patient-controlled analgesia was continued during the 24 h postoperative period. The frequency and dose of fentanyl (μg/kg) during the postoperative period were calculated from PCA pump records and normalized to body weight. The intensity of pain sensation was assessed at 3 and 24 h postoperatively using a 100 mm VAS, with 0 mm indicating no pain and 100 mm indicating the worst pain imaginable.

### Genotyping

Either whole blood or oral mucosa samples were collected from all subjects for the genetic analysis. DNA was extracted from whole blood or oral mucosa samples using the conventional phenol-chloroform method or QIAamp DNA Mini Kit (Qiagen K.K., Tokyo, Japan) according to the manufacturer’s instructions.

Genotyping STRs in the *CNR1*, *GRIN2A*, and *PENK* genes was performed by Aoba Genetics, Inc., (Yokohama, Japan). Approximately 15 ng of genomic DNA from each participant was used as the template for genotyping using polymerase chain reaction (PCR) with fluorescent dye-labeled primers and an ABI PRISM 3130xl Genetic Analyzer (Applied Biosystems Japan, Tokyo, Japan). Briefly, PCRs were performed with a 5′-VIC-labeled specific forward primer for each STR and Invitrogen AccuPrime *Taq* DNA polymerase, High Fidelity (Thermo Fisher Scientific K.K., Tokyo, Japan), under the following conditions: 94°C for 2 min, 35 cycles of 94°C for 30 s, 57°C for 30 s, and 68°C for 30 s, followed by 68°C for 7 min. The specific primers for STRs in the *CNR1*, *GRIN2A*, and *PENK* genes were the following: *CNR1* STR (forward, 5′-GCT​GCT​TCT​GTT​AAC​CCT​GC-3′; reverse, 5′-TAC​ATC​TCC​GTG​TGA​TGT​TCC-3′), *GRIN2A* STR (forward, 5′-GAA​GGA​AGC​ATG​TGG​GAA​ATG​CAG-3′; reverse, 5′-GTT​TCT​TGC​TGG​GTA​CAG​TTA​TCC​CCC​T-3′), *PENK* STR forward, 5′-TAA​TAA​AGG​AGC​CAG​CTA​TG-3′; reverse, 5′-ACA​TCA​GAT​GTA​AAT​GCA​AGT-3′; Chan et al., 1994; Comings et al., 1997; Itokawa et al., 2003). The nucleotide lengths of the PCR products were determined using an ABI PRISM 3130xl Genetic Analyzer and GeneMapper 4.0 software (Applied Biosystems Japan). To evaluate the actual repeat numbers of the STRs, direct sequencing was performed with PCR products that were amplified from samples whose repeat numbers were determined to be homozygous by GeneMapper (Supplementary Material). The cycle sequencing reaction with the BigDye Terminator 3.1 Cycle Sequencing Kit (Applied Biosystems Japan) was performed according to the manufacturer’s instructions, and the reaction products were purified. Nucleotide sequences of the products, including the STRs, were determined using an ABI PRISM 3100 Genetic Analyzer (Applied Biosystems Japan).

Genotyping for the 68 bp VNTR in the *PDYN* gene was previously described (Zimprich et al., 2000). Briefly, PCRs were performed with the primers 5′-AGC​AAT​CAG​AGG​TTG​AAG​TTG​GCA​GC-3′ and 5′-GCA​CCA​GGC​GGT​TAG​GTA​GAG​TTG​TC-3′ under the following thermal cycle conditions: 95°C for 2 min, 35 cycles of 95°C for 30 s, 70°C for 30 s, and 72°C for 1 min, followed by 72°C for 8 min. The 68 bp repeat number of each sample was determined by electrophoretic mobility of the PCR product in agarose gel electrophoresis.

### Statistical Analysis

All of the statistical analyses were performed using SPSS Statistics 24 software (IBM Japan Ltd., Tokyo, Japan). All of the clinical data, including the participants’ demographic data and analgesic requirements of opioids after open abdominal surgery and orthognathic cosmetic surgery, were first analyzed for normal probability distributions and homoscedastic variance using the Kolmogorov-Smirnov test and *F*-test, respectively. These clinical parameters did not have a normal probability distribution; thus, subsequent statistical analyses were performed using nonparametric methods. Associations between the participants’ demographic characteristics and clinical data, including peripheral pain sensation and analgesic requirements of opioids, were examined using the Mann-Whitney *U* test. Associations between variances of STRs or VNTRs and clinical data were statistically analyzed using the Spearman’s rank correlation test. Multiple-testing corrections were performed using Holm’s method to adjust *p* values that were derived from multiple comparisons in the association analyses. Values of *p* < 0.05 were considered statistically significant.

## Results

The present study included three association analyses with clinical data on peripheral pain sensation and analgesic requirements of opioids after open abdominal surgery and orthognathic cosmetic surgery (Table 1). The distributions of the participants’ demographic data, including age, body weight, and body mass index (BMI), were different between the association analyses. The distribution of the age of patients who underwent open abdominal surgery was significantly different from participants whose peripheral pain sensation was assessed and patients who underwent orthognathic cosmetic surgery (U = 1,363.50 and U = 219.50, respectively, *p* < 0.001, Mann-Whitney *U* test). The distribution of the participants’ ages in the study of peripheral pain sensation was significantly associated with latencies to cold and mechanical pain perception (Spearman’s rank correlation coefficient *ρ* = 0.324, *p* < 0.001, and *ρ* = 0.185, *p* < 0.05, respectively). The latency to mechanical pain perception were significantly different between sexes (U = 2306.50, *p* < 0.001, Mann-Whitney *U* test). The latency to cold pain perception was associated with mechanical pain perception (*ρ* = 0.366, *p* < 0.001). The distribution of ages of patients who underwent open abdominal surgery was significantly associated with the frequency of analgesic use during the 24 h postoperative period (*ρ* = −0.168, *p* < 0.05). The body weight and BMI distributions for patients who underwent orthognathic cosmetic surgery were significantly associated with 24 h postoperative fentanyl dose (*ρ* = −0.164, *p* < 0.01, and *ρ* = −0.144, *p* < 0.05, respectively).

**TABLE 1:** Patients’ demographic and clinical data.

Demographic data	Clinical data
Volunteers:	
Number of patients (male/female)	192 (121/71)
Age (years)	36.14 ± 12.69 (20–65)
Peripheral pain sensation-related traits:	
Latency to cold pain perception (s)	23.75 (17.13–36.88); 6.00–180.00
Latency of mechanical pain perception (kg)	2.30 (1.79–3.02); 0.77–5.31
Patients who underwent open abdominal surgery:	
Number of patients (male/female)	139 (81/58)
Age (years)	63.63 ± 9.96 (28–80)
Body weight (kg)	56.52 ± 10.44 (35–80)
BMI (kg/m^2^)	22.47 ± 3.50 (14.50–35.03)
Analgesic requirements of opioid-related traits:	
Frequency of analgesic use during the 24 h postoperative period	0 (0–1); 0–6
24 h postoperative analgesic dose converted to systemic pentazocine (mg)	0.00 (0.00–15.00); 0.00–135.00
24 h postoperative analgesic dose (including epidural analgesics) converted to systemic pentazocine (mg)	127.50 (96.00–177.00); 36.00–315.00
Patients who underwent orthognathic cosmetic surgery:	
Number of patients (male/female)	252 (89/163)
Age (years)	25.58 ± 7.44 (15–50)
Body weight (kg)	58.22 ± 11.23 (38–128)
BMI (kg/m^2^)	21.36 ± 3.02 (14.88–37.10)
Analgesic requirements of opioid-related traits:	
Frequency of fentanyl use during the 24 h postoperative period	6 (3–12); 0–111
24 h postoperative fentanyl dose (μg/kg)	2.32 (1.14–4.08); 0.00–13.82
VAS pain score at 3 h (mm)	25 (13–48); 0–90
VAS pain score at 24 h (mm)	24 (10–40); 0–83

The data are expressed as numbers, mean ± standard deviation (range), or median (interquartile range); range.

We focused on STRs in the *CNR1*, *GRIN2A*, and *PENK* genes and VNTRs in the *PDYN* gene and examined whether STRs and VNTRs in these genes were associated with peripheral pain sensation and analgesic requirements of opioids. Short tandem repeats in the *CNR1*, *GRIN2A*, and *PENK* genes were AAT trinucleotide, GT dinucleotide, and CA dinucleotide repeat polymorphisms, respectively. The VNTR in the *PDYN* gene was 68 bp repeats (Table 2). These repeat polymorphisms widely varied with many alleles (repeat number) and genotypes (repeat number/repeat number). Two alleles of STRs and VNTRs in each subject were defined as shorter (S) and longer (L) alleles according to their repeat numbers in each patient. Associations between STRs or VNTRs and the clinical data were analyzed according to the S and L alleles of each STR.

**TABLE 2:** Short tandem repeats analyzed in the present study.

Gene	Coding protein	dbSNP rs#	Position	Location in gene	STR/VNTR
*CNR1*	cannabinoid receptor 1	rs72426053, rs1562491880, rs1244069759, rs1164873271	6q15	3′ flanking region	(AAT)n
*GRIN2A*	Ionotropic glutamate receptor NMDA type subunit 2A	rs3219790	16p13.2	5′ flanking region	(GT)n
*PENK*	proenkephalin	rs3138832	8q12.1	3′ flanking region	(CA)n
*PDYN*	prodynorphin	rs1568548100, rs869080647, rs1568548112, rs1568548137, rs1568548201	20p13	5′ flanking region	(68 bp)n

The distribution of the STR in the *CNR1* gene showed a large repeat number and wide variation as 9–18 repeats (6-7 variations) in the both S and L alleles (Table 3). In contrast, although the repeat number of the STR in the *GRIN2A* gene was also as large as 20–35 (14 variations), the distributions of repeat numbers were different between the S and L alleles (S allele: 20–29 repeats; L allele: 20–35 repeats). Variations of the STR in the *PENK* gene and VNTR in the *PDYN* gene were as low as five variations (11–15 repeats) and four variations (1–4 repeats), respectively. Associations between STRs or VNTRs and clinical data on peripheral pain sensation, including latencies to cold and mechanical pain perception, were examined using the Spearman’s rank correlation test after the correction for age and sex. The repeat number of the L allele of the *CNR1* STR was slightly associated with the latency to mechanical pain perception in male volunteers (Spearman’s rank correlation coefficient *ρ* = 0.186, *p* = 0.043), but this association was not confirmed after multiple-testing correction using the Holm method for each STR.

**TABLE 3:** Associations between S and L alleles of STRs and peripheral pain sensation.

		*CNR1*		*GRIN2A*		*PENK*		*PDYN*	
		S allele	L allele	S allele	L allele	S allele	L allele	S allele	L allele
Number of repeats:Number of alleles (allelic frequency in S and L alleles)		9: 43 (0.224)	9: 1 (0.005)	20: 25 (0.130)	20: 1 (0.005)	12: 31 (0.161)	12: 1 (0.005)	1: 13 (0.068)	
		12: 22 (0.115)	12: 1 (0.005)	21: 9 (0.047)	21: 2 (0.010)	13: 91 (0.474)	13: 32 (0.167)	2: 171 (0.891)	2: 141 (0.734)
		13: 9 (0.047)		22: 20 (0.104)	22: 5 (0.026)	14: 68 (0.354)	14: 155 (0.807)	3: 8 (0.042)	3: 48 (0.250)
		14: 48 (0.250)	14: 15 (0.078)	23: 27 (0.141)	23: 3 (0.016)		15: 2 (0.01)		4: 3 (0.016)
		15: 54 (0.281)	15: 80 (0.417)	24: 28 (0.146)	24: 12 (0.063)				
		16: 13 (0.068)	16: 75 (0.391)	25: 65 (0.339)	25: 66 (0.344)				
		17: 1 (0.005)	17: 18 (0.094)	26: 9 (0.047)	26: 29 (0.151)				
				27: 6 (0.031)	27: 42 (0.219)				
				28: 1 (0.005)	28: 17 (0.089)				
				29: 2 (0.010)	29: 7 (0.036)				
					31: 5 (0.026)				
					32: 1 (0.005)				
					34: 1 (0.005)				
				35: 1 (0.005)					
Latency to cold pain perception	ρ	−0.047	0.027	0.003	−0.035	0.019	−0.041	0.079	−0.019
	*p*	0.520	0.713	0.963	0.632	0.796	0.571	0.277	0.790
Latency to mechanical pain perception	ρ	−0.077	0.125	−0.036	0.015	−0.021	−0.048	0.011	-0.115
	*p*	0.293	0.086	0.622	0.837	0.774	0.511	0.878	0.112

Spearman’s rank correlation tests were conducted to examine associations between shorter (S) and longer (L) alleles in each STR or VNTR and the latency to pain perception. *ρ*: Spearman’s rank correlation coefficient.

Repeat numbers of STRs and VNTRs in patients who underwent open abdominal surgery showed a similar distribution as participants whose peripheral pain sensation was assessed (Table 4). The surgical procedures for open abdominal surgery mostly included gastrectomy for gastric cancer and colectomy for colorectal cancer. The frequency of analgesic use during the 24 h postoperative period (frequency of analgesic use), 24 h postoperative analgesic dose (converted to systemic pentazocine; analgesic dose), and 24 h postoperative analgesic dose, including epidural analgesics (analgesic dose including epidural analgesics) were analyzed as an index for analgesic requirements of opioids in this association analysis. The repeat variation of the S allele of the *GRIN2A* STR was significantly associated with analgesic dose, including epidural analgesics (Spearman rank correlation coefficient *ρ* = −0.200, *p* = 0.018), but this association was not confirmed after multiple-testing correction using the Holm method for each STR.

**TABLE 4:** Associations between S and L alleles of STRs and postoperative analgesia in patients who underwent open abdominal surgery.

		*CNR1*		*GRIN2A*		*PENK*		*PDYN*	
		S allele	L allele	S allele	L allele	S allele	L allele	S allele	L allele
Number of repeats:Number of alleles (allelic frequency in S and L alleles)		9: 19 (0.137)	9: 3 (0.022)	20: 15 (0.108)	20: 3 (0.022)	11: 1 (0.007)		1: 17 (0.122)	
		10: 1 (0.007)		21: 11 (0.079)	21: 3 (0.022)	12: 22 (0.158)	12: 2 (0.014)	2: 119 (0.856)	2: 98 (0.705)
		11: 1 (0.007)		22: 15 (0.108)	22: 2 (0.014)	13: 59 (0.424)	13: 21 (0.151)	3: 3 (0.022)	3: 36 (0.259)
		12: 14 (0.101)	12: 1 (0.007)	23: 15 (0.108)	23: 4 (0.029)	14: 57 (0.410)	14: 110 (0.791)		4: 5 (0.036)
		13: 8 (0.058)	13: 1 (0.007)	24: 27 (0.194)	24: 12 (0.086)		15: 6 (0.043)		
		14: 28 (0.201)	14: 10 (0.072)	25: 39 (0.281)	25: 34 (0.245)				
		15: 49 (0.353)	15: 45 (0.324)	26: 11 (0.079)	26: 24 (0.173)				
		16: 13 (0.094)	16: 60 (0.432)	27: 6 (0.043)	27: 24 (0.173)				
			17: 12 (0.086)		28: 16 (0.115)				
			18: 1 (0.007)		29: 6 (0.043)				
					30: 3 (0.022)				
					31: 4 (0.029)				
					32: 2 (0.014)				
					33: 1 (0.007)				
					34: 1 (0.007)				
Frequency of analgesic use	ρ	−0.153	−0.140	-0.093	0.015	0.107	−0.010	−0.066	0.029
	*p*	0.078	0.109	0.275	0.859	0.211	0.903	0.437	0.735
Analgesic dose	ρ	−0.134	−0.121	−0.104	−0.005	0.099	−0.005	−0.009	0.046
	*p*	0.123	0.165	0.221	0.953	0.246	0.954	0.915	0.592
Analgesic dose including epidural analgesics	ρ	0.068	−0.050	−0.200	0.009	0.050	0.028	−0.048	0.011
	*p*	0.440	0.566	0.018	0.914	0.557	0.745	0.575	0.900

Spearman’s rank correlation tests were conducted to examine associations between shorter (S) and longer (L) alleles in each STR or VNTR and the frequency of analgesic use during the 24 h postoperative period (frequency of analgesic use), 24 h postoperative analgesic dose converted to systemic pentazocine (analgesic dose), and 24 h postoperative analgesic dose, including epidural analgesics (analgesic dose, including epidural analgesics). *ρ*: Spearman’s rank correlation coefficient.

Repeat numbers of the STRs and VNTRs in patients who underwent orthognathic cosmetic surgery showed a similar distribution as participants whose peripheral pain sensation was assessed (Table 5). The index for analgesic requirements of opioids, including the frequency of fentanyl use during the 24 h postoperative period (frequency of fentanyl use), 24 h postoperative fentanyl dose (fentanyl dose), VAS pain score at 3 h, and VAS pain score at 24 h, were analyzed. The repeat number of the L allele of the *PENK* STR was significantly associated with VAS pain score at 24 h (Spearman’s rank correlation test; *ρ* = 0.143, *p* = 0.023), but no significant association was found after Holm’s multiple-testing correction. The repeat number of the S allele of the *CNR1* STR was significantly associated with the frequency of fentanyl use, fentanyl dose, and VAS pain score at 3 h (*ρ* = 0.199, *p* = 0.002, *ρ* = 0.174, *p* = 0.006, and *ρ* = 0.135, *p* = 0.033, respectively). Associations with the frequency of fentanyl use and fentanyl dose were also statistically significant after Holm’s multiple-testing correction.

**TABLE 5:** Associations between S and L alleles of STRs and postoperative analgesia in patients who underwent orthognathic cosmetic surgery.

		*CNR1*		*GRIN2A*		*PENK*		*PDYN*	
		S allele	L allele	S allele	L allele	S allele	L allele	S allele	L allele
Number of repeats:Number of alleles (allelic frequency in S and L alleles)		9: 41 (0.163)	9: 2 (0.008)	18: 1 (0.004)		12: 34 (0.135)		1: 9 (0.083)	
		12: 30 (0.119)	12: 3 (0.012)	19: 1 (0.004)		13: 127 (0.504)	13: 39 (0.155)	2: 94 (0.870)	2: 68 (0.630)
		13: 8 (0.032)		20: 28 (0.111)	20: 1 (0.004)	14: 90 (0.357)	14: 209 (0.829)	3: 4 (0.037)	3: 35 (0.324)
		14: 55 (0.218)	14: 27 (0.107)	21: 17 (0.067)	21: 1 (0.004)		15: 3 (0.012)		4: 5 (0.046)
		15: 96 (0.381)	15: 76 (0.302)	22: 28 (0.111)	22: 5 (0.020)				
		16: 20 (0.079)	16: 125 (0.496)	23: 42 (0.167)	23: 3 (0.012)				
		17: 1 (0.004)	17: 17 (0.067)	24: 43 (0.171)	24: 19 (0.075)				
			18: 1 (0.004)	25: 61 (0.242)	25: 91 (0.361)				
				26: 18 (0.071)	26: 46 (0.183)				
				27: 11 (0.044)	27: 43 (0.171)				
				28: 1 (0.004)	28: 20 (0.079)				
					29: 7 (0.028)				
					30: 3 (0.012)				
					31: 2 (0.008)				
					32: 7 (0.028)				
					33: 2 (0.008)				
					35: 1 (0.004)				
Frequency of fentanyl use	ρ	0.199 *	−0.006	−0.070	−0.020	0.021	0.081	−0.152	−0.079
	*p*	0.002	0.922	0.268	0.750	0.746	0.200	0.117	0.415
Fentanyl dose	ρ	0.174 *	−0.016	−0.103	−0.044	0.053	0.070	−0.132	−0.118
	*p*	0.006	0.802	0.106	0.486	0.408	0.269	0.173	0.226
VAS pain score at 3 h	ρ	0.135	0.036	0.003	0.078	−0.067	0.085	−0.025	−0.060
	*p*	0.033	0.569	0.957	0.219	0.293	0.181	0.796	0.540
VAS pain score at 24 h	ρ	0.059	−0.061	0.000	−0.076	0.015	0.143	−0.031	−0.103
	*p*	0.354	0.340	0.994	0.230	0.815	0.023	0.752	0.289

Spearman’s rank correlation tests were conducted to examine associations between shorter (S) and longer (L) alleles in each STR or VNTR and the frequency of fentanyl use during the 24 h postoperative period (frequency of fentanyl use), 24 h postoperative fentanyl dose (fentanyl dose), VAS pain score at 3 h, and VAS pain score at 24 h. ρ: Spearman’s rank correlation coefficient. *p < 0.05, significant difference between repeat number of STR and postoperative analgesia after Holm’s multiple-testing correction.

## Discussion

The present study was performed with clinical data on peripheral pain sensation and analgesic requirements of opioids in patients who underwent open abdominal surgery and orthognathic cosmetic surgery. The frequency of fentanyl use during the 24 h postoperative period in patients who underwent orthognathic cosmetic surgery showed a wide distribution in the range of 0–111 (Table 1). The lockout time of the PCA pump was set at 10 min. Thus, patients could receive fentanyl at most 144 times during the 24 h postoperative period. The VAS scores of the patient who received fentanyl 111 times were 5 and 25 at 3 and 24 h postoperatively, respectively. The duration of anesthesia and surgery for this patient was 185 min (interquartile range: 156, 185) and 120 min (interquartile range: 90, 119), respectively. This patient was included in the study because this patient met all of the inclusion criteria and none of the exclusion criteria. In the participants’ demographic data, age was positively associated with latencies to cold and mechanical pain perception (Spearman’s rank correlation coefficient *ρ* = 0.324, *p* < 0.001, and *ρ* = 0.185, *p* < 0.05, respectively). Thus, pain sensitivity was considered to decrease with age. The age of the patients who underwent open abdominal surgery was also negatively associated with the frequency of analgesic use (Spearman rank correlation coefficient *ρ* = −0.168, *p* = 0.049). The lower frequency of analgesic use would be caused by lower pain sensitivity in older patients. The latency to mechanical pain perception in females was lower than in males (Mann-Whitney *U* = 6284.50, *p* < 0.001), but no difference in cold pain perception was found between sexes, indicating that females are more sensitive to mechanical pain but not too cold pain compared with males (data not shown). The latency to cold pain perception was measured by the cold-pressor test immediately before surgery in patients who underwent orthognathic cosmetic surgery. In patients who underwent open abdominal surgery, the latencies to cold and mechanical pain perception were not examined. The cold-pressor test was slightly different from the peripheral pain perception test that was performed in volunteers (Fukuda et al., 2009). The distribution of the latency to cold pain perception in patients who underwent orthognathic cosmetic surgery was 2.0–150 s (median: 14.0, interquartile range: 9.0, 23.0) and was slightly associated with fentanyl dose and VAS pain score at 24 h (Spearman rank correlation coefficient *ρ* = −0.128, *p* = 0.042, and *ρ* = −0.126, *p* = 0.047, respectively), but these associations were not confirmed after multiple-testing correction using the Holm method to analyze the latency to cold pain perception with four clinical parameters, including the frequency of fentanyl use, fentanyl dose, VAS pain score at 3 h, and VAS pain score at 24 h. The distribution of the latency to cold pain perception in patients who underwent orthognathic cosmetic surgery was not associated with the patients’ ages (*ρ* = −0.022, *p* = 0.734). The low standard deviation of the patients’ ages (mean ± standard deviation: 25.58 ± 7.44 years; Table 1) suggested that patients who underwent orthognathic cosmetic surgery and enrolled in the study were within a narrow age range and may be expected to be uniform with regard to pain perception.

Variations of repeat numbers were different between three STRs and VNTRs in the present study. The repeat numbers and variations of STRs in the *CNR1*, *GRIN2A*, and *PENK* genes were 9–18 repeats (10 variations), 18–35 repeats (18 variations), and 11–15 repeats (five variations), respectively. On the contrary, the repeat number and variation of the VNTR in the *PDYN* gene was 1-4 repeats and four variations. The repeat numbers of STRs in the *CNR1*, *GRIN2A*, and *PENK* genes were large, rather than the VNTR in the *PDYN* gene. Among these STRs, high variations of repeat numbers of STRs were found in the *CNR1* and *GRIN2A* genes rather than the *PENK* gene, indicating that not all STR variations are high. Replication slippage events outnumber point mutations by one or two orders of magnitude in STRs (Pumpernik et al., 2008). Mutations that are caused by DNA slippage in STRs occurred without a minimal threshold length, and mutation rates decreased and increased with the repeat length and total length of STRs, respectively (Leclercq et al., 2010), which can support the difference in repeat number variations between STRs in the *CNR1*, *GRIN2A*, and *PENK* genes. However, mutation rates in STRs converged at the upper limit (0.001–0.01) at over 20 nucleotides of the total length of STRs, although the repeat length and differences in STR variations (e.g., repeat number and total length of STRs) could be influenced by other factors, such as chromosomal background near the STRs. The STR in the *CNR1* gene showed multiple peaks in the distribution of repeat numbers at 9–12 and 15 repeats. This unusual allelic distribution of the STR in the *CNR1* gene was described in previous studies, which suggests our data on allelic distribution in the *CNR1* gene was not a genotyping error (Comings et al., 1997; Li et al., 2000; Siegfried et al., 2004; Ruiz-Contreras et al., 2013). The present study was conducted with different races and ethnicities, indicating that the multiple peaks of allelic frequency of the STR in the *CNR1* gene was not attributable to racial or ethnic differences in allelic frequency. Such multiple peaks of the allelic frequency of STRs were also shown at another microsatellite loci, DYS385a/b in the Y chromosome, although allelic frequencies of most STRs had a single peak (Kayser et al., 2000). Considering these studies together, the mutation rate in the *CNR1* STR would change at multiple stages with the length of the STR, which would be a structural characteristic of the STR in the *CNR1* gene.

Short tandem repeats have been reported to be associated with gene expression and human traits (Jakubosky et al., 2020). Approximately 2,000 significant expression STRs colocalized with regulatory elements and were considered to modulate certain histone modifications (Gymrek et al., 2016). These expression STRs also contributed 10–15% of the heritability that was mediated by all common variants and were enriched in various clinical conditions that were reported by genome-wide association studies. Expression-mediating VNTRs also exerted a strong influence on the expression of proximal genes, and 80% of these VNTRs have a maximum effect size of at least 0.3 (Bakhtiari et al., 2021). Short tandem repeats and VNTRs in regulatory elements of genes can influence gene expression. The influence of each allele of STRs and VNTRs on gene expression would be expected to be different according to lengths of STRs and VNTRs. If the elongation of an STR is recessive for a phenotype, then the phenotype would be attributable to the S allele of the STR. Conversely, if the elongation of an STR is dominant for a phenotype, then the phenotype would be attributable to the L allele of the STR. Investigating STRs and VNTRs according to the S and L alleles rather than according to groups that are divided by the threshold of the length of STRs and VNTRs effectively assesses the genetic vulnerability to a phenotype. In the present study, the S allele of the AAT trinucleotide STR in the *CNR1* gene was positively associated with the frequency and dose of postoperative fentanyl use in patients who underwent orthognathic cosmetic surgery, indicating that analgesic effects of opioids would be low in patients with long AAT repeats. The elongation of AAT repeats of the STR in the *CNR1* gene would be recessive for the analgesic effects of opioids in patients who underwent orthognathic cosmetic surgery. The protein expression of CNR1 in lymphocytes from patients with long AAT repeats of the STR in the *CNR1* gene (≥12 repeats in both alleles) was significantly lower than in patients with short AAT repeats (homozygous or heterozygous for alleles with ≤11 repeats; Rossi et al., 2013). This report also indicated that the long repeat of the STR in the *CNR1* gene would be recessive for phenotypes that are related to the *CNR1* gene. AAT STRs are the most representative trinucleotide repeats in the human genome, but they are less frequent in exons (Kozlowski et al., 2010). AAT STRs are mainly located in the 3′-untranslated region (UTR). The 3′UTRs of genes are related to the stability of mRNA, and extension of the 3′UTR generally causes lower stability and expression of mRNA. The lower protein expression of CNR1 in carriers of long AAT repeats of the *CNR1* STR may be caused by the lower stability of *CNR1* mRNA.

Short tandem repeats in the *CNR1* gene were reported to be associated with the vulnerability to irritable bowel syndrome (Park et al., 2011; Jiang et al., 2014), multiple sclerosis (Ramil et al., 2010; Rossi et al., 2011, 2013), schizophrenia (Ujike et al., 2002; Martínez-Gras et al., 2006), and substance use disorders (Comings et al., 1997; Zhang et al., 2004; Ballon et al., 2006). The T2-weighted lesion load was inversely correlated with gray matter volume in the left frontal and cingulate cortex and right temporal cortex in multiple sclerosis patients with long AAT repeats of the *CNR1* STR (Rossi et al., 2013). The previous study indicated the possibility that some gray matter regions that are related to the analgesic effects of opioids would be vulnerable to inflammatory stimulation in carriers of long AAT repeats of the *CNR1* STR. Additionally, CNR1 colocalized with μ-opioid receptors in the same presynaptic nerve terminals in the nucleus accumbens, caudate putamen, and dorsal horn in rats (López-Moreno et al., 2010). A positive allosteric modulator of CNR1 signaling enhanced morphine antinociception in mice (Slivicki et al., 2020). These results indicate the critical role of the endogenous cannabinoid system *via* CNR1 in modulating the analgesic effects of opioids for orofacial surgery.

Genetic contributions to analgesic effects of opioids are influenced by psychological and environmental factors, including race, ethnicity, culture, and pain sensation status. To further elucidate genetic variability of the STR in the *CNR1* gene that contributes to analgesic effects of opioids, replication studies will be required in different races/ethnicities with sufficient sample sizes for each effect size. Additionally, elucidating the molecular mechanisms that underlie the association between repeat polymorphisms, including STRs and VNTRs, and analgesic effects of opioids may lead to a precise understanding of individual differences in analgesic effects of opioids and improve treatment strategies for pain.

## Data Availability

The datasets presented in this study can be found in online repositories. The names of the repository/repositories and accession number(s) can be found in the article/Supplementary Material.
