# Supplementary material for: Short Tandem Repeat Variation in the CNR1 Gene Associated With Analgesic Requirements of Opioids in Postoperative Pain Management
Source: Front Genet. 2022 Mar 3;13:815089. doi: 10.3389/fgene.2022.815089 (PMC8963810; doi:10.3389/fgene.2022.815089)
Supplement: Supplementary file 1 [file DataSheet2.docx]

(AAT)n STR in the *CNR1* gene

ID#59 (patient who underwent orthognathic cosmetic surgery)

>TTGCTGCTTCTGTTAACCCTGCCATTAAGGGAAAGAGGTGTAATCTTTATTATTATTATTATTATTATTATTATTATTATTATTATTATTATACTTTAAGTTTTAGGGTACATGTGCACAACATGCAGGTTAGTTACATATGTATACATGTGCCATGTTGGTGTGCTGCATGTTCTCACTCATAGGTGGGAAT

ID#62

>GCCATTAAGGGAAAGAGGTGTAATCTTTATTATTATTATTATTATTATTATTATTATTATTATTATTATTATACTTTAAGTTTTAGGGTACATGTGCACAACATGCAGGTTAGTTACATATGTATACATGTGCCATGTTGGTGTGCTGCATGTTCTCACTCATAGGTGGGAAT

ID#73

>TGCTTCTGTTAACCCTGCCATTAAGGGAAAGAGGTGTAATCTTTATTATTATTATTATTATTATTATTATTATTATTATTATACTTTAAGTTTTAGGGTACATGTGCACAACATGCAGGTTAGTTACATATGTATACATGTGCCATGTTGGTGTGCTGCATGTTCTCACTCATAGGTGGGAAT

#74

>GCTGCTTCTGTTAACCCTGCCATTAAGGGAAAGAGGTGTAATCTTTATTATTATTATTATTATTATTATTATTATTATTATTATTATACTTTAAGTTTTAGGGTACATGTGCACAACATGCAGGTTAGTTACATATGTATACATGTGCCATGTTGGTGTGCTGCATGTTCTCACTCATAGGTGGGAAT

#225

>TTGCTGCTTCTGTTAACCCTGCCATTAAGGGAAAGAGGTGTAATCTTTATTATTATTATTATTATTATTATTATTATTATTATTATACTTTAAGTTTTAGGGTACATGTGCACAACATGCAGGTTAGTTACATATGTATACATGTGCCATGTTGGTGTGCTGCATGTTCTCACTCATAGGTGGGAAT

#242

>TGCTTCTGTTAACCCTGCCATTAAGGGAAAGAGGTGTAATCTTTATTATTATTATTATTATTATTATTATTATTATTATTATTATTATACTTTAAGTTTTAGGGTACATGTGCACAACATGCAGGTTAGTTACATATGTATACATGTGCCATGTTGGTGTGCTGCATGTTCTCACTCATAGGTGGGAAT

(GT)n STR in the *GRIN2A* gene

#67

>ATCAGAGGAGTGGGTTCCCGTACGGGGGTGGCGAGGCACAGGGAGAAGCATTGGAGCAGACTCTGTGTGTGTGTGTGTGTGTGTGTGTGTGTGTGTGTGTGTGTGTGTGTGTCGGCGCGCCTGTG

#68

>ATCAGAGGAGTGGGTTCCCGTACGGGGGTGGCGAGGCACAGGGAGAAGCATTGGAGCAGACTCTGTGTGTGTGTGTGTGTGTGTGTGTGTGTGTGTGTGTGTGTGTGTGTGTGTGTCGGCGCGCCTGTG

#79

>ATCAGAGGAGTGGGTTCCCGTACGGGGGTGGCGAGGCACAGGGAGAAGCATTGGAGCAGACTCTGTGTGTGTGTGTGTGTGTGTGTGTGTGTGTGTGTGTGTGTGTGTGTGTGTGTGTCGGCGCGCCTGTG

#80

>ATCAGAGGAGTGGGTTCCCGTACGGGGGTGGCGAGGCACAGGGAGAAGCATTGGAGCAGACTCTGTGTGTGTGTGTGTGTGTGTGTGTGTGTGTGTGTGTGTGTGTGTGTGTGTCGGCGCGCCTGTG

#193

>ATCAGAGGAGTGGGTTCCCGTACGGGGGTGGCGAGGCACAGGGAGAAGCATTGGAGCAGACTCTGTGTGTGTGTGTGTGTGTGTGTGTGTGTGTGTGTGTGTGTGTGTGTCGGCGCGCCTGTG

#208

>ATCAGAGGAGTGGGTTCCCGTACGGGGGTGGCGAGGCACAGGGAGAAGCATTGGAGCAGACTCTGTGTGTGTGTGTGTGTGTGTGTGTGTGTGTGTGTGTGTGTGTGTGTGTGTGTGTCGGCGCGCCTGTG

#250

>ATCAGAGGAGTGGGTTCCCGTACGGGGGTGGCGAGGCACAGGGAGAAGCATTGGAGCAGACTCTGTGTGTGTGTGTGTGTGTGTGTGTGTGTGTGTGTGTGTGTGTGTGTGTGTGTCGGCGCGCCTGTG

(CA)n STR in the *PENK* gene

#61

>AATGTATTTTCACTCTCATTTCTGGAAATCAGATACATGAAGAAAAACCAAAGCCTAATAAAGGAGCCAGCTATGCCCCCATTAAACACACACACACACACACACACACACACATACTTGCATTTACATCAGATGTGTATGTCTCTTTTCTTCAGTAAAGTATTTTAGAAGTTAGTAAGAAATTAAAGATAATTTAGTCCAATTTTACAAATGAAG

#69

>AATGTATTTTCACTCTCATTTCTGGAAATCAGATACATGAAGAAAAACCAAAGCCTAATAAAGGAGCCAGCTATGCCCCCATTAAACACACACACACACACACACACACACATACTTGCATTTACATCAGATGTGTATGTCTCTTTTCTTCAGTAAAGTATTTTAGAAGTTAGTAAGAAATTAAAGATAATTTAGTCCAATTTTACAAATGAAG

#71

>AATGTATTTTCACTCTCATTTCTGGAAATCAGATACATGAAGAAAAACCAAAGCCTAATAAAGGAGCCAGCTATGCCCCCATTAAACACACACACACACACACACACACACATACTTGCATTTACATCAGATGTGTATGTCTCTTTTCTTCAGTAAAGTATTTTAGAAGTTAGTAAGAAATTAAAGATAATTTAGTCCAATTTTACAAATGAAG

#72

>AATGTATTTTCACTCTCATTTCTGGAAATCAGATACATGAAGAAAAACCAAAGCCTAATAAAGGAGCCAGCTATGCCCCCATTAAACACACACACACACACACACACACACACATACTTGCATTTACATCAGATGTGTATGTCTCTTTTCTTCAGTAAAGTATTTTAGAAGTTAGTAAGAAATTAAAGATAATTTAGTCCAATTTTACAAATGAAG

#73

>AATGTATTTTCACTCTCATTTCTGGAAATCAGATACATGAAGAAAAACCAAAGCCTAATAAAGGAGCCAGCTATGCCCCCATTAAACACACACACACACACACACACACACATACTTGCATTTACATCAGATGTGTATGTCTCTTTTCTTCAGTAAAGTATTTTAGAAGTTAGTAAGAAATTAAAGATAATTTAGTCCAATTTTACAAATGAAG

#75

>AATGTATTTTCACTCTCATTTCTGGAAATCAGATACATGAAGAAAAACCAAAGCCTAATAAAGGAGCCAGCTATGCCCCCATTAAACACACACACACACACACACACACACACACACTTGCATTTACATCAGATGTGTATGTCTCTTTTCTTCAGTAAAGTATTTTAGAAGTTAGTAAGAAATTAAAGATAATTTAGTCCAATTTTACAAATGAAG

#78

>AATGTATTTTCACTCTCATTTCTGGAAATCAAGATACATTGAAGAAAAACCAAAGCCTAATAAAGGAGCCAGCTATGCCCCCATTAAACACACACACACACACACACACACACACATACTTGCATTTACATCAGATGTGTATGTCTCTTTTCTTCAGTAAAGTATTTTAGAAGTTAGTAAGAAATTAAAGATAATTTAGTCCAATTTTACAAATGAAG

#94

>AATGTATTTTCACTCTCATTTCTGGAAATCAGATACATGAAGAAAAACCAAAGCCTAATAAAGGAGCCAGCTATGCCCCCATTAAACACACACACACACACACACACACACATACTTGCATTTACATCAGATGTGTATGTCTCTTTTCTTCAGTAAAGTATTTTAGAAGTTAGTAAGAAATTAAAGATAATTTAGTCCAATTTTACAAATGAAG
